# Microbiota-Derived Extracellular Vesicles as Potential Mediators of Gut–Brain Communication in Traumatic Brain Injury: Mechanisms, Biomarkers, and Therapeutic Implications

**DOI:** 10.3390/biom15101398

**Published:** 2025-09-30

**Authors:** Tarek Benameur, Abeir Hasan, Hind Toufig, Maria Antonietta Panaro, Francesca Martina Filannino, Chiara Porro

**Affiliations:** 1 Department of Biomedical Sciences, College of Medicine, King Faisal University, Al-Ahsa 31982, Saudi Arabia; 2 Department of Surgery, College of Medicine, King Faisal University, Al-Ahsa 31982, Saudi Arabia; 3 Department of Biosciences, Biotechnologies and Environment, University of Bari, I-70125 Bari, Italy; 4 Department of Clinical and Experimental Medicine, University of Foggia, I-71100 Foggia, Italy

**Keywords:** microbiota-derived extracellular vesicles, traumatic brain injury, gut, homeostasis, biomarkers, precision medicine, artificial intelligence, multi-omics, neuroinflammation, blood–brain barrier, gut microbiota–brain axis

## Abstract

Traumatic brain injury (TBI) remains a major global health problem, contributing significantly to morbidity and mortality worldwide. Despite advances in understanding its complex pathophysiology, current therapeutic strategies are insufficient in addressing the long-term cognitive, emotional, and neurological impairments. While the primary mechanical injury is immediate and unavoidable, the secondary phase involves a cascade of biological processes leading to neuroinflammation, blood–brain barrier (BBB) disruption, and systemic immune activation. The heterogeneity of patient responses underscores the urgent need for reliable biomarkers and targeted interventions. Emerging evidence highlights the gut–brain axis as a critical modulator of the secondary phase, with microbiota-derived extracellular vesicles (MEVs) representing a promising avenue for both diagnosis and therapy. MEVs can cross the intestinal barrier and BBB, carrying biomolecules that influence neuronal survival, synaptic plasticity, and inflammatory signaling. These properties make MEVs promising biomarkers for early detection, severity classification, and prognosis in TBI, while also offering therapeutic potential through modulation of neuroinflammation and promotion of neural repair. MEV-based strategies could enable tailored interventions based on the individual’s microbiome profile, immune status, and injury characteristics. The integration of multi-omics with artificial intelligence is expected to fully unlock the diagnostic and therapeutic potential of MEVs. These approaches can identify molecular subtypes, predict outcomes, and facilitate real-time clinical decision-making. By bridging microbiology, neuroscience, and precision medicine, MEVs hold transformative potential to advance TBI diagnosis, monitoring, and treatment. This review also identifies key research gaps and proposes future directions for MEVs in precision diagnostics and gut microbiota-based therapeutics in neurotrauma care.

## 1. Introduction

TBI is a major global public health concern, with no effective clinical treatment strategies addressing the long-term consequences on patients’ health and quality of life. It results from external forces such as those caused by motor vehicle accidents, armed conflict, violence, terrorist attacks, falls, or sports-related impacts [1]. TBI is a complex, multiphase condition characterized by dynamic interactions between the brain, the peripheral organs, and the immune system [2,3]. Previously reported estimates indicated that the annual global incidence of TBI varies widely, from 27 to 69 million cases [4,5]. More recent data highlight the alarming global burden that TBI has reached, affecting all age groups, with an estimated 5.48 million years lived with disability [6].

TBI poses significant healthcare challenges, including diagnosis, effective treatment, and long-term rehabilitation. The condition’s complex pathophysiology and the wide variability in how individuals respond to injury affect the effective management [7,8,9,10,11].

Furthermore, TBI, regardless of severity, be it mild, moderate, or severe, has been strongly linked to significant long-term cognitive impairment. In the chronic phase, patients with TBI continue to experience persistent cognitive deficits, which are closely associated with both the initial severity of injury and the cumulative burden of trauma [12].

TBI can lead to a wide range of functional impairments, which often contribute to considerable post-traumatic disability affecting physical, cognitive, emotional, behavioral, and sensorimotor domains. Although significant progress has been made in unraveling the cascade of events involved in TBI pathophysiology, many of the underlying mechanisms remain incompletely understood [13,14]. Advances in diagnostic tools such as neuroimaging, blood-based biomarkers, and genomic profiling have enhanced the ability to characterize TBI; however, patient outcomes continue to show considerable variability [15]. In this review, we will discuss the major challenges in diagnosing TBI, the limitations of current therapeutic approaches, and the ongoing difficulties faced by caregivers throughout the patient’s recovery process. Recent lines of evidence have highlighted several novel approaches. Among these, extracellular vesicles (EVs) have emerged as crucial mediators of intercellular communication. EVs and their associated cargo are gaining recognition as promising biomarkers for TBI, as they mirror cellular states and injury responses [16]. Beyond their diagnostic potential, EVs are key mediators of intercellular communication in the nervous system, particularly between neurons and glial cells, which may significantly influence TBI progression.

In the same context, the gut microbiota has gained considerable attention as a key modulator of the gut–brain axis, particularly in the regulation of inflammation associated with various neurological conditions, including TBI [17]. The bidirectional communication between the CNS and the gut microbiota is mediated by intricate immunological, endocrine, metabolic, and neural pathways [18,19,20]. Disruption of this communication is commonly referred to as dysbiosis and has been linked to altered neuroinflammatory responses and impaired neurological recovery following brain injury.

A promising area of investigation involves MEVs, which have emerged as critical mediators in neurological conditions, with growing evidence supporting their involvement in neurodegenerative diseases and their potential therapeutic relevance in TBI [19].

Within the gut–brain axis, EVs are increasingly recognized for their role in modulating neuronal signaling, neuroinflammatory response, and metabolism. MEVs can cross the BBB and carry microbial signals that influence immune and neuronal function. Their ability to reflect the gut’s microbial environment makes them a strong candidate for early diagnosis, while their therapeutic potential lies in modulating neuroinflammation and enhancing tissue repair and treatment.

In this review, we will have a glance at the latest advances, highlighting the emerging alternatives that aim to improve clinical outcomes and long-term care for patients with TBI. The growing insights into the complex interplay between the gut and brain have highlighted MEVs as emerging and promising diagnostic and therapeutic tools. By mediating gut–brain signaling, MEVs hold considerable potential to facilitate more targeted, personalized, and effective interventions in the management of TBI, thereby constituting the main focus of this review.

## 2. Traumatic Brain Injury and the Gut Microbiota–Brain Axis

### 2.1. TBI-Induced Changes in Microbial Composition and Diversity

The interrelationship between gut microbiota composition, TBI severity, and the systemic metabolomic profiles was investigated in a rat model of TBI before and after the induced injury by utilizing pre- and post-injury sampling. The study revealed statistically significant variations in both gut bacterial population levels and their associated metabolites following injury [21]. Specifically, a reduction in the abundance of approximately 14 bacterial genera was observed, while 12 bacterial genera showed an increased proliferation. Notably, the relative abundance of the phylum Bacteroidetes decreased from 22.7% prior to injury to 19.8% seven days post-TBI, whereas Firmicutes increased from a baseline level of 66.1% to 68.0%over the same period following TBI.

Further analysis of fecal samples demonstrated a significant decrease in microbiota α-diversity and a marked shift in overall microbial community structure following injury, indicating a substantial post-TBI dysbiosis. Interestingly, despite these pronounced compositional and functional changes in the gut microbiota, the study found no statistically significant correlation between post-injury microbial profiles and the severity of TBI. These findings suggest that gut microbial alterations occur independently of injury magnitude and may represent a generalized host response to neural trauma rather than a direct consequence of injury severity [21].

### 2.2. Microbiota Modulate Both Systemic and Central Immune Responses Post-Injury

Following TBI, the gut–brain axis interconnection pathways play a critical role in the modulation of both systemic and central immune responses. Although not yet fully elucidated, several mechanisms have been investigated for their impact on these complex neuroimmune modulations. These include TBI-induced gut dysbiosis associated with blood–brain barrier (BBB) alterations, activation of the inflammasome and microglia, neuronal dysfunction, and infiltration of inflammatory cells into the injured tissue.

One well-characterized mechanism involves increased BBB permeability resulting from exposure to translocated microbial metabolites and neurotransmitters produced by the gut microbiota. Metabolites such as tryptophan derivatives, butyrate, and short-chain fatty acids (SCFAs), along with neurotransmitters including γ-aminobutyric acid (GABA) and serotonin (5-HT), have been shown to influence BBB integrity. Experimental studies in germ-free (GF) animal models demonstrated that recolonization with specific bacterial strains, such as Clostridium tyrobutyricum, enhances the production of anti-inflammatory metabolites like butyrate. These metabolites help maintain gut barrier integrity and upregulate the tight junction proteins occludin and claudin-5 in the BBB, thereby restoring its function. Furthermore, inoculating GF mice with SCFA-producing bacterial strains has been shown to re-establish BBB integrity [22].

More recently, the role of uncontrolled activation of an innate immune component, the inflammasome, has emerged as a key contributor to post-TBI neuroinflammation and systemic immune responses. Several studies have highlighted its diagnostic and therapeutic potential in neurological disorders such as multiple sclerosis and Alzheimer’s disease (AD). Inflammasome activation can be triggered extracellularly via pathogen- or danger-associated molecular patterns (PAMPs/DAMPs), or intracellularly through signals such as lysosomal damage and mitochondrial reactive oxygen species (ROS). Among the various inflammasome complexes, the NLRP3 inflammasome is the most extensively studied. Upon activation, it promotes the conversion of pro-caspase-1 to active caspase-1, which in turn facilitates the maturation of proinflammatory cytokines IL-18 and IL-1β.

The gut microbiota can initiate inflammasome activation both locally and centrally through the release of microbial metabolites that act as signaling molecules. Bile acids, taurine, and histamine, among other microbiota-derived metabolites, have been shown to modulate inflammasome activity. These metabolites may reach the CNS via humoral pathways or through afferent signaling via the vagus nerve. While inflammasome activation serves a protective role by initiating appropriate immune responses following injury, excessive or uncontrolled activation can result in pyroptosis, a proinflammatory form of programmed cell death, thereby exacerbating neuronal damage [22,23].

### 2.3. The Gut–Brain Axis (GBA)-MEVs: A Bidirectional Communication Pathway

The role of gut microbiome in modulating general health and well-being is widely studied, revealing marked convergence with the aging process and pathophysiology of various diseases, including neuroinflammatory disorders. Central to this interaction is GBA, a complex dynamic and bidirectional signaling pathway of communication between the CNS and the gut. While the precise mechanisms governing this crosstalk remain incompletely understood, a growing body of research implicates multiple neuroendocrine and immunological pathways. These include vagal nerve signaling pathways, the hypothalamic–pituitary axis, in addition to the recently emerged microbiota extracellular vesicles (MEVs). The interaction between these systems is essential for maintaining gut homeostasis, as well as modulating emotional behaviours and cognitive functions [24]. The gut microbiota has emerged as a key modulator of the GBA. Animal studies have demonstrated that gut bacterial colonization is fundamental for the proper development of both the central and enteric nervous systems [25]. Gut microbiota was found to produce a range of metabolites and neurotransmitters such as short-chain fatty acids (SCFAs), tryptophan precursors and metabolites, 5-hydroxytryptamine (5-HT), gamma-aminobutyric acid (GABA), and lipopolysaccharides (LPSs), all of which exert regulatory effects on neuronal, microglial, and immune cell function [26].

Disruption of gut homeostasis triggers the release of SCFA, which can cross the BBB and influence brain development and immune function, although the mechanisms are not yet fully elucidated. SCFAs have been shown to promote microglial maturation and activation that results in shaping the neuronal circuits and modulating the CNS immunity.

The treatment of cultured microglia with acetate was able to suppress inflammation by reducing the expression of proinflammatory cytokines (IL-1β, IL-6, and TNF-α) and decreasing the phosphorylation of p38 MAPK, JNK, and NF-κB. Furthermore, SCFAs modulate neurotrophic factors crucial for neuronal survival, growth, and plasticity, including nerve growth factor (NGF), glial cell line-derived neurotrophic factor (GDNF), and brain-derived neurotrophic factor (BDNF), which are involved in learning, memory, and neurodegenerative disease mechanisms.

SCFAs play a vital role in regulating the key neurotransmitters [27,28]. Recent research investigating the bidirectional GBA signaling pathways in irritable bowel syndrome (IBS) patients undergoing cognitive behavioural therapy (CBT) showed that respondents to CBT exhibited significantly reduced levels of both microbiota and tryptophan, the serotonin precursor, suggesting a link between gut microbial modulation and therapeutic outcomes [29]. Additional evidence highlights the neuroprotective role of microbial metabolites. For example, indole-3-propionic acid (IPA), a tryptophan metabolite produced by *Clostridium sporogenes* and *Peptostreptococcus anaerobius*, has been associated with elevated serum BDNF and reduced TNF-α levels in elderly individuals [29]. Furthermore, germ-free animal models consistently show reduced BDNF expression compared to conventional controls, underscoring the importance of gut microbiota in regulating neurotrophic signaling [30].

Neurotransmitters such as γ-aminobutyric acid (GABA), dopamine (DA), norepinephrine (NE), serotonin (5-HT), and histamine play crucial roles in CNS/gut microbiota interactions. The vagus nerve acts as a bidirectional conduit for signals mediated by these neurotransmitters, integrating input from the CNS and gut through the autonomic nervous system (ANS), enteric nervous system (ENS), HPA axis, and immune pathways. This integrated signaling governs not only cognitive and behavioral processes but also gut motility, immune regulation, and inflammatory responses. Certain gut microbes, such as Lactobacillus rhamnosus JB-1, have demonstrated the ability to modulate GABA receptor expression in the brain, leading to anxiolytic and antidepressant effects, which hold potential for microbiota-based adjunct therapies [29,30].

The immunomodulatory effects of the gut microbiome on the CNS function have been widely postulated. Gut bacterial wall components, particularly LPS, can traverse the BBB and activate microglial cells via TLR2 and TLR4, which are expressed both centrally and in enteric neurons. This triggers a proinflammatory cascade characterized by increased production of inflammatory cytokines such as TNF-α, IL-6, IL-1β, IFN-γ, and proinflammatory mediators like COX-2 and NOS-2 in both the gut and the brain. Notably, germ-free animals exhibit reduced microglial responsiveness to LPS, further supporting the microbiota’s role in CNS immune regulation [30]. Furthermore, Lee et al. demonstrated that administration of the probiotic *Bifidobacterium longum* NK46 in 5XFAD transgenic and aged mice reduced gut-derived LPS and inhibited NF-κB activation. This led to improvements in tight junction integrity in the colon, decreased neuroinflammation, elevated BDNF expression in the hippocampus, and alleviated cognitive decline [31].

### 2.4. The Impact of TBI on Gut Microbiota

The interconnection between TBI and the gut microbiota dysbiosis has been thoroughly investigated, with growing evidence supporting the hypothesis that the disruption of the gut–brain axis following TBI may significantly alter the composition, abundance, and functions of gut microbiota. Emerging research suggests that this dysregulation can lead to the production of neurotoxic metabolites such as secondary bile acids, trimethylamine-N-oxide (TMAO), tryptophan derivatives, and indoles that can cross the BBB, triggering neuroinflammation and neurodegenerative changes that contribute to psychological and cognitive impairment. However, the underlying mechanisms remain to be fully elucidated [32,33].

In another related study, Zheng et al. explored the effect of TBI on gut microbiota dysbiosis in a mouse model using 16s rRNA gene sequencing and untargeted metabolomics profiling. They revealed that TBI is associated with incremental gut bacteria dysbiosis and metabolomics. Their findings revealed that TBI induces progressive gut microbial dysbiosis and alters tryptophan metabolism, potentially sustaining the upregulation of Lyz2 (a gene encoding lysozyme), microglial activation, and subsequent neuroinflammation [34].

## 3. From Primary Injury to Neurodegeneration: Mechanistic Insight into TBI

TBIs are further classified as primary or secondary injuries depending on the underlying pathophysiological mechanisms. Primary TBIs occur at the time of trauma. These injuries disrupt cellular membranes and ionic homeostasis, which significantly contribute to axonal injury and degeneration, edema, loss of energy, a shift to anaerobic metabolism, and acidosis. Secondary TBIs, on the other hand, are the outcome of many interconnected pathophysiological mechanisms, including BBB disruption, neuroinflammation, oxidative stress, mitochondrial dysfunction, excitotoxicity, and neuronal death, that emerge minutes, hours, or days after the primary injury and critically influence outcomes [35,36]. The dysregulation of BBB integrity results in an increased vascular permeability, exudation of plasma and plasma-derived proteins, cerebral edema, increased intracranial pressure, and infiltration by immune cells. Several mechanisms contribute to BBB dysregulation post-TBI. A recent study demonstrated that TBI downregulates claudin-5, a critical tight junction protein of the BBB. This reduction is associated with the elevated TGF-β1 and increased permeability of the BBB. The consequent disruption facilitates the recruitment and activation of the inflammatory cells and upregulation of leukocyte adhesion molecules, leading to infiltration of neutrophils, macrophages, and lymphocytes.

These activated cells release proinflammatory cytokines, reactive oxygen species (ROS), and lysosomal enzymes, further disrupting the BBB and amplifying inflammation [36]. Microglia, the resident innate immune cells of the CNS, play a central role in post-TBI neuroinflammation, pathogenesis, and neurodegeneration. Upon activation, they trigger inflammatory signaling pathways and cytokine release, including IL-1β [23]. Notably, chronic microglial activation has been observed up to 17 years post-TBI, as evidenced by increased PK11195 binding to microglial benzodiazepine receptors. Similarly, persistent microglial and macrophage activation was found in professional football players a decade after retirement, preceding the onset of cognitive decline [37]. In TBI mouse models, microglial depletion with PLX5622 significantly downregulated interferon-regulated and inflammatory genes (C1qc, Tlr2, Mecp2, Cybb, and S100b), as well as neuropathology-associated genes (Sncb, Cntnap2, Scn1a, and Ddc), while also altering astrocytic and neuronal gene expression, indicating that microglia influence gene regulation in other CNS cell types as well [37,38].

Supporting these findings, Lin Cai et al. (2022) [38] demonstrated that reducing microglial activation with ACT001, a guaianolide sesquiterpene lactone, improved motor function and BBB integrity in a TBI model, making microglia a therapeutic target in mitigating TBI-induced pathology.

Neurodegenerative changes following secondary TBIs emerge within the first week and progress over months to years, driven by neuroinflammation, cellular activation, and oxidative stress, underlying TBI-associated neurodegeneration and offer potential therapeutic targets [39]. Individuals in the chronic phase often experience persistent cognitive deficits, which correlate strongly with the initial severity and cumulative burden, rather than with the rate of cognitive decline over time [12]. Emerging evidence also suggests that interpersonal variability may influence cognitive outcomes, especially in mild TBI patients. These variations should be carefully considered when developing strategies for the management of post-TBI cognitive impairment [40].

## 4. Microbiota-Derived Extracellular Vesicles (MEVs)

Based on the nature of their outer membrane, bacteria are broadly classified into Gram-negative (G−) and Gram-positive (G+). G− bacteria possess a complex double-plasma membrane separated by the periplasmic space. In contrast, G+ bacteria lack an outer membrane and instead have a thick peptidoglycan layer surrounding the cytoplasmic membrane [29]. This fundamental structural difference not only defines bacterial classification but also shapes the way in which they generate extracellular vesicles (EVs), leading to their categorization as G− or G+ derived.

Building on these structural features, it is well documented that microbes, like all living cells, spontaneously produce extracellular vesicles (EVs) [41]. Both G− and G+ bacteria, whether pathogenic or non-pathogenic, release membrane vesicles, often referred to as bacterial EVs or microbiota-derived (MEVs). They typically have a diameter of 40–400 nm and vary in type and biogenesis.

G- bacteria produce several types of membrane-derived extracellular vesicles (MEVs), including outer membrane vesicles (OMVs), inner–outer membrane vesicles (O-IMVs), and explosive outer membrane vesicles (E-OMVs). OMVs are spherical particles that originate via outer membrane blebbing and are composed of an outer leaflet rich in lipopolysaccharides (LPSs) and an inner leaflet of phospholipids. These vesicles encapsulate a variety of outer membrane and periplasmic components [42,43]. O-IMVs, which incorporate both outer and inner membrane elements, are produced through a blebbing mechanism involving the simultaneous protrusion of the cytoplasmic and outer membranes, often associated with peptidoglycan layer weakening by autolysins. Consequently, O-IMVs contain inner membrane proteins and cytosolic contents in addition to periplasmic constituents [44,45]. Another form of vesicle, the E-OMV “explosive outer membrane vesicles”, results from explosive cell lysis, a process typically triggered by phage-derived endolysins that enzymatically degrade the peptidoglycan layer, leading to sudden cellular disintegration and the release of vesicles laden with cytoplasmic material, including chromosomal DNA [46,47,48].

In G- bacteria, two primary biogenesis pathways govern MEV production: membrane blebbing and explosive cell lysis [49]. Blebbing is driven by specific physicochemical and structural factors, including lipid asymmetry in the outer membrane, where LPS are predominantly located on the external leaflet and phospholipids on the internal leaflet. This asymmetry contributes to membrane curvature, promoting vesicle formation [50]. Disruption in the peptidoglycan–outer membrane linkage, either due to reduced protein cross-linking or altered transmembrane protein conformations, further facilitates vesicle release [51]. Several genes have been implicated in maintaining this structural integrity, including OprI, OmpA, Pal, and TolA in *Pseudomonas aeruginosa*; TolA, TolQ, and Tol/Pal in Escherichia coli; OmpA in Acinetobacter baumannii; and the ABC transporter complex VacJ/YrbC in Hemophilus influenzae and Vibrio cholerae, with its homologue Mla identified in *E. coli* [52]. Deletion or downregulation of these genes reduces outer membrane–peptidoglycan interactions, enhancing vesiculation [50,53].

Another proposed mechanism of vesicle biogenesis involves the accumulation of misfolded proteins or peptidoglycan fragments in the periplasm, generating turgor pressure that drives membrane protrusion. In *Pseudomonas aeruginosa*, the quorum-sensing molecule PQS (Pseudomonas quinolone signal) in the outer membrane can destabilize lipid packing, enhancing curvature and vesicle release [54,55,56,57]. Additionally, in some Gram-negative bacteria, vesicle formation is linked to flagellar assembly: membrane vesicles arise along the flagellar sheath and are shed during rotation, providing another route of release [58].

While OMVs and O-IMVs originate via membrane blebbing, E-OMVs and EO-IMVs (explosive inner–outer membrane vesicles) are the products of lytic processes. The latter vesicles are released during phage-mediated lysis, a violent event wherein cellular contents, including chromosomal DNA and inner membrane components, are discharged into the extracellular milieu due to the rapid collapse of the bacterial cell envelope [47,48].

In G+ bacteria, cytoplasmic membrane vesicles (CMVs) have been identified. Unlike OMVs, they lack LPS and periplasmic elements due to the absence of an outer membrane, but they carry peptidoglycan, lipids, proteins, and nucleic acids, thus reflecting similar functional roles of OMVs [59]. CMV biogenesis can occur through a lytic process termed “bubble cell death,” in which cells extrude membrane material through pores in the peptidoglycan layer, producing explosive CMVs (ECMVs). Although the cytoplasmic membrane remains partly intact, vesicle release ultimately compromises cell integrity, leaving ghost or dead cells [60,61].

As in G- systems, CMV release in G+ bacteria can be triggered by phage-derived endolysins. Moreover, endogenous autolysins, normally involved in daughter cell separation during division, can also drive vesicle release under conditions of stress, while peptidoglycan hydrolases and β-lactam antibiotics further stimulate CMV biogenesis in this context [62].

### 4.1. Composition of MEVs

MEVs are lipid bilayer-enclosed nanoparticles released by gut microbiota and other microbial communities whose composition depends on the bacterial species, vesicle biogenesis pathway, and environmental conditions [63,64]. Functionally, MEVs serve as pivotal mediators of intercellular communication, facilitating a range of biological effects through the delivery of molecular cargo.

This cargo typically comprises proteins, lipids, nucleic acids, and small metabolites, each contributing to the vesicle’s functional repertoire. Protein content reflects their structural complexity and functional specialization. These proteins include structural components, porins, ion channels, transporters, enzymes, and stress-response elements. MEVs derived from G- bacteria are particularly enriched in outer membrane proteins, including OmpA, OmpC, and OmpF, critical for membrane integrity and transport [59,65].

These vesicles contain periplasmic proteins such as AcrA and alkaline phosphatase, along with virulence factors, inner membrane and cytoplasmic proteins, and biofilm-related proteins. Structural components, including LPS, phospholipids, and peptidoglycan (10–20%), further characterize these vesicles [66]. Strain-specific proteins, particularly in commensal and probiotic G- strains, support bacterial colonization, competition, and persistence in the intestinal environment [67,68]. In contrast, MEVs from G+ bacteria show a distinct molecular profile, rich in cytoplasmic and membrane proteins, but with elevated peptidoglycan (>50%) and lipoteichoic acid [69].

Pathogenic G+ vesicles often harbor toxins and virulence factors, whereas probiotic bacteria-derived vesicles contain proteins that promote immunomodulation and beneficial host–microbe interactions [70]. Lipids are crucial for MEV structure, stability, and formation. In G- bacteria, outer membrane vesicles (OMVs) often contain lipid species absent or less abundant in the outer membrane, such as glycerophospholipids, phosphatidylglycerol, phosphatidylethanolamine, and cardiolipin, which contribute to membrane fluidity and stability [71]. LPS, another key component, often differs from the membrane form, with altered sugar moieties and deacylated lipid A, affecting host immune recognition and inflammatory signaling [72].

In G+ bacteria, cytoplasmic membrane vesicles (CMVs) exhibit a distinct lipid profile, typically enriched in unsaturated and medium-chain fatty acids, phosphatidylethanolamine, sphingolipids, and triacylglycerols. Lipid composition is strain-dependent and responsive to environmental conditions, reflecting adaptive strategies for vesicle formation and host interaction [59,73]. Beyond lipids, MEVs serve as vectors for genetic material, facilitating horizontal gene transfer and delivering nucleic acids to host cells, where they can modulate immune responses [74,75]. Encapsulated DNA often encodes genes associated with antibiotic resistance, virulence, and stress adaptation, including toxins such as α-toxin and perfringolysin O [7,76]. MEVs also carry RNA, protected from degradation and efficiently delivered into eukaryotic cells [77]. These primarily small non-coding RNAs function similarly to eukaryotic microRNAs, regulating gene expression post-transcriptionally and modulating host immune responses and cellular phenotypes [78,79].

### 4.2. Role of MEVs in Host–Microbe Communication

MEVs can cross the epithelial barriers and disseminate through both lymphatic and circulatory systems, occurring not only under pathological conditions with compromised intestinal barrier integrity but also in healthy mucosal tissues [80]. Their route across the intestinal epithelium varies by bacterial species and involves both phagocytic and non-phagocytic mechanisms [81]. G- OMVs, a major MEVs subtype, cross the intestinal epithelium via the paracellular (between cells) or transcellular (through cells) routes, reaching sites beyond the gastrointestinal tract [82,83,84]. Once in the lamina propria, OMVs are phagocytosed by immune cells and have been detected in systemic fluids such as blood and urine.

MEV passage is most evident in patients with intestinal barrier dysfunction, where pathogenic OMVs can disrupt the intestinal barrier by altering the permeability of cellular junctions [79,80]. Additionally, OMVs from gut microbiota also act at a distance from their parent cells, delivering enzymes and bioactive molecules. These include proteases and glycosidases that degrade polysaccharides, mucin sulfatases, and inositol polyphosphatases that break down dietary phytate to release phosphate and related derivatives [85]

MEVs interact with host cells through multiple pathways, including transcellular and paracellular migration, macropinocytosis, clathrin- and caveolin-mediated endocytosis, and pattern recognition receptor engagement. Dendritic cells internalize OMVs via TLR2, influencing immune responses such as regulatory T cell induction, while macrophages also contribute to uptake [86,87].

The presence of bacterial DNA in human serum indicates that MEVs can cross the intestinal epithelium into the bloodstream [88,89]. Circulating microbial DNA reflects the diversity of gut microbiota, showing that MEVs carry genetic material representative of their parent bacteria and can disseminate systemically from the intestinal lumen and diffuse into the systemic circulation.

Once in the bloodstream, MEVs can affect distant organs, including the brain. Although the BBB is a highly selective barrier, evidence shows that MEVs can reach the CNS and trigger signaling pathways that modulate neural function [90,91]. Carrying proteins, small non-coding RNAs, and other bioactive molecules, MEVs influence diverse brain cell types [92]. Gut–brain communication via MEVs occurs mainly through systemic circulation and the autonomic nervous system, with additional input from immune mechanisms.

The circulatory route: MEVs cross the intestinal epithelium into the bloodstream, reaching peripheral organs and the CNS. After crossing the BBB, they can modulate neurotransmitters such as serotonin, dopamine, norepinephrine, and GABA [93]. They also transport precursors, hormone-like metabolites, and SCFAs that regulate neuronal and microglial activity by binding to CNS receptors.

In parallel, MEVs act via the autonomic nervous system, especially the enteric nervous system and vagus nerve. By interacting with vagal receptors, they modulate motility and stimulate signaling molecules such as SCFAs and cholecystokinin [94,95,96,97].

The immune system provides a third route: OMVs are internalized by dendritic cells via a TLR2- and actin-dependent mechanism, inducing cytokines such as IL-22, IL-17, and IL-10. These immune signals affect the CNS indirectly through neural and HPA axis activation or directly by crossing the BBB [98,99].

### 4.3. Microbiota-Derived Extracellular Vesicles in Neurodegeneration

The pathogenesis of neurological disorders has been mainly attributed to intrinsic factors of the CNS. However, increasing evidence supports the pivotal role of the gut microbiota in neurodevelopment and brain function [100,101]. MEVs, although not recognized as a direct causative agent of neurodegenerative disorders, actively contribute to the microbiota–gut–brain axis, exerting both harmful and protective effects. MEVs, including outer membrane vesicles (OMVs), mediate communication and carry diverse bioactive molecules capable of influencing neurological pathways. Below, we summarize recent findings on the role of MEVs in Alzheimer’s and Parkinson’s diseases, which are linked to TBI.

Alzheimer’s disease (AD) is a progressive neurodegenerative disease characterized by cognitive decline and memory impairment, β-amyloid (Aβ) plaque deposition, and intracellular hyperphosphorylated tau (p-Tau) forming neurofibrillary tangles, leading to synaptic dysfunction, neuroinflammation, and neuronal loss [102,103].

Emerging evidence highlights the role of gut microbiota-derived extracellular vesicles (MEVs) in AD pathogenesis [104]. In AppNL-G-F AD mice, raised under germ-free conditions, exhibited reduced amyloid-β pathology and synaptic deficits, effects reversed by administration of MEVs from commensal gut microbiota, demonstrating their influence on microglial activation and plaque accumulation [105].

Pathogenic bacterial EVs also contribute to AD progression. *Helicobacter pylori*-derived EVs can cross the BBB, activate astrocytes and microglia, and exacerbate Aβ pathology and cognitive decline via C3-C3aR signaling. Inhibiting C3aR blocks these effects, preserving synaptic and cognitive function [106]. Similarly, oral MEVs released from *Porphyromonas gingivalis*, a key pathogen in periodontitis, carry virulence factors such as gingipains and LPS. These MEVs can trigger systemic inflammation and cognitive deficits resembling memory impairment. Mechanistically, they upregulate proinflammatory mediators (e.g., TNF-α, Iba1-associated GP, LPS, and NF-κB) while downregulating key neuroprotective elements, including BDNF receptors, claudin-5, and NMDA receptors. Importantly, these microbiota-derived EVs can translocate into the brain via the trigeminal nerve and periodontal vasculature, linking oral dysbiosis to neuroinflammation and AD progression [107].

Further research by Gong et al. (2022) [108] supported the role of MEVs in AD. Indeed, P. gingivalis (PG)-derived EVs impair memory and learning ability in mice. In addition, they reduce the expression of the hippocampal tight junction proteins (ZO-1), occludin, and claudin-5, and activate both astrocytes and microglia, leading to IL-1β release, tau, and NLRP3 inflammasome activation [108].

Kayo Yoshida et al. found that these MEVs translocated to the brain, where Yoshida et al. showed that these MEVs translocate to the brain, where gingipains induce proinflammatory cytokine expression in glial cells [109]. Beyond oral pathogens, gut microbiota also influence AD via bile acid metabolism. Wei-Leng Chin et al. reported that Aβ accumulation reduces *Lactobacillus johnsonii* and increases *Clostridium* spp., promoting toxic bile acid production. Notably, *L. johnsonii*-derived EVs suppressed *C. scindens* growth and lithocholic acid synthesis, highlighting the small intestine microbiota and their EVs as critical modulators of AD pathology [110].

These findings underscore not only the critical role of MEVs in AD but also in other neurodegenerative diseases such as PD. PD is characterized by dopaminergic neuron loss in the substantia nigra and motor symptoms such as tremor, bradykinesia, and rigidity, with abnormal α-synuclein (α-syn) aggregation as the pathological hallmark [111]. Gastrointestinal dysfunction often precedes PD motor symptoms, supporting the gut-origin hypothesis where α-syn aggregates in the enteric nervous system or olfactory bulb and spreads to the CNS via the vagus nerve, potentially mediated by EVs [112,113].

MEVs also influence PD progression. Probiotic Lactobacillus plantarum-derived EVs reduce LPS-induced inflammation by downregulating TNF-α and IL-6, suggesting neuroprotective effects [114]. Conversely, *E. coli*-derived EVs from individuals carrying PD-associated LRRK2 variants deliver curli bacterial amyloid proteins to colonic epithelial cells, enhancing DAPK1 expression and phosphorylated α-syn, promoting neurodegeneration [115,116]. Curli-producing bacteria such as Streptococcus, Staphylococcus, Salmonella, and Klebsiella, Citrobacter, and Bacillus may similarly contribute to PD pathology, highlighting MEVs as both mediators of disease and potential therapeutic targets [113].

Taken together, MEVs have a dual effect on neurodegeneration, with commensal-derived vesicles offering protection while pathogen- or dysbiosis-derived vesicles exacerbate AD and PD pathology through neuroinflammation, protein aggregation, and neuronal dysfunction. Since TBI is a major risk factor for both AD and PD, sharing mechanisms like BBB disruption, chronic microglial activation [117], and MEV-mediated neuroinflammation, it further connects these conditions along a common gut–brain axis. This is reinforcing the role of the gut–brain axis as a critical regulator of neurodegenerative progression in TBIs and reinforcing the argument that MEV-driven mechanisms observed in various neurodegenerative conditions are highly relevant to TBI.

Table 1 summarizes recent findings on the effects of MEVs from selected commensal and pathogenic bacteria, which are increasingly implicated in the pathogenesis of neurodegenerative disorders, including AD and PD.

## 5. The Emerging Role of the Microbiota-Derived Extracellular Vesicles in TBI

### 5.1. Microbiota Derived Extracellular Vesicles in Post-TBI Neuroinflammation: Gut–Immune–Brain Interactions

MEVs are emerging mediators of intercellular and host–microbiota communication, delivering bioactive molecules that modulate immune responses to injury. They play crucial roles in systemic immunity and neuroprotection, yet their functions in post-TBI remain poorly understood. MEVs carry important signaling molecules that can modulate complications following TBI [118]. As discussed above, TBI initiates a cascade of neuroinflammatory responses that can result in long-term cognitive and neurological deficits. Recent evidence highlights the pivotal role of the gut microbiota and its derived EVs in modulating these responses, suggesting significant involvement of the gut–immune–brain axis in TBI pathophysiology, diagnosis, and recovery. MEVs can cross the intestinal barrier, enter the bloodstream, and reach the brain, where they interact with the CNS-resident immune cells and neurons to influence neuroinflammatory pathways. These EVs have been shown to modulate microglial activation and other CNS immune responses, contributing to the regulation of neuroinflammation. Interestingly, MEVs were shown to contribute to TBI pathophysiology by promoting endothelial dysfunction, vascular leakage, and neuroinflammation through HMGB1-mediated activation of RAGE/Cathepsin B signaling and NLRP3 inflammasome pathways, suggesting their potential as therapeutic targets and biomarkers for secondary brain injury [119]. From the point of view of mechanistic, MEVs can alter cytokine production, modulate microglial activation states, and affect BBB permeability; by delivering microbial antigens or signaling molecules, they activate pattern recognition receptors on host immune cells, triggering the release of proinflammatory cytokines and chemokines and potentially facilitating peripheral immune cell infiltration into the brain [34].

Although the precise mechanisms by which MEVs cross the BBB remain not fully understood, recent research has shown the effect of MEVs on the brain and provided clear evidence of their neuroinvasive potential. Labeling techniques and functional assays in murine models exposed to probiotics, human BBB cell models, and postmortem analyses of AD brains have demonstrated that MEVs can traverse the BBB and influence neural environments [24,120]. These findings underscore the relevance of MEVs in gut–brain axis communication and highlight their potential as vectors of microbial signals affecting CNS function in various contexts, including TBI and the associated complex neurodegenerative disorders.

MEVs may exert a dual effect, either beneficial or deleterious, depending on the context of their release. Further research is warranted to elucidate their precise mechanism of action in TBI, ideally using animal models equipped with advanced tracking systems to monitor the biodistribution and functional impact of these MEVs in vivo.

Understanding these processes opens new avenues for therapeutic interventions, including strategies to modulate the gut microbiota or block the transfer of microbial EVs to the CNS, which may mitigate neuroinflammatory responses and improve post-TBI outcomes. Further research is needed to identify the specific components of microbiota-derived EVs responsible for these effects and to develop targeted therapies to harness their neuroprotective potential.

### 5.2. Extracellular Vesicles in Post-TBI Immune Modulation and Therapy: Emerging Evidence

MEVs modulate brain immune responses after TBI in both stimulatory and suppressive ways, depending on their molecular cargo. LPS-enriched MEVs activate toll-like receptors (TLRs), particularly TLR4, on innate immune cells, driving proinflammatory cytokine release and amplifying neuroinflammation following TBI [99]. In addition to TLR signaling, MEVs can also trigger cytosolic receptors such as NLRs, with peptidoglycan from *Helicobacter pylori* and *Escherichia coli*, enhancing inflammatory signaling. In contrast, commensal bacteria like *Bacteroides fragilis* produce polysaccharide-rich MEVs that activate dendritic cells and drive regulatory T cell (Treg) differentiation, fostering the production of anti-inflammatory cytokines and suppressing neuroinflammation [99].

Recently, MEVs have gained considerable interest as potential therapeutic agents in TBI. Various mechanisms have been proposed to explain how microbiota or their derived products, such as MEVs, can confer neuroprotection. For instance, Jing, Y et al. have demonstrated that fecal microbiota transplantation (FMT) in mice with spinal cord injury (SCI) resulted in improved locomotor function, re-establishment of descending motor pathways, enhanced neuronal survival, and synaptic restoration. These effects were attributed to microbiota-derived SCFAs and inhibition of the IL-1β/NF-κB signaling pathway, correlating with observed anti-inflammatory responses and post-FMT SCI improvement [121].

Probiotics-derived extracellular vesicles (PEVs) have an emerging role in reversing gut bacterial dysbiosis, restoring the homeostasis of gut microbiota, and intestinal health. PEVs from beneficial bacteria such as *Lacticaseibacillus rhamnosus*, *Lactobacillus plantarum*, and *Bacteroides acidifaciens* exert immunomodulatory effects in the gut. Oral administration of PEVs in animal models led to downregulation of key proinflammatory cytokines, including TNF-α, IL-1β, and IL-6, and suppression of the expression of key inflammatory genes such as TLR4, Myd88, P53, and NF-κB.

In addition to their local effects in the gut, PEVs can cross both the intestinal barrier and the BBB, thereby potentially exerting systemic and neuroprotective effects. Several mouse studies have suggested that PEVs derived from *Lacticaseibacillus rhamnosus*, *Lacticaseibacillus paracasei*, and *Lactiplantibacillus plantarum* reduce neuroinflammatory responses in a dose-dependent manner in preclinical models such as the injured brain tissues. These effects include attenuation of inflammation, promotion of neuronal regeneration, and improvement in cognitive deficits. However, the specific molecular components within PEVs responsible for these therapeutic outcomes remain to be fully investigated and characterized [122].

Additionally, Huang et al. have reported a significant neurological improvement in SCI mice following infusion with extracellular vesicles derived from epidural fat mesenchymal stem cells (EF-MSCs) [123]. The beneficial outcomes were attributed to the significant downregulation of the NLRP3 inflammasome and upregulation of anti-apoptotic protein Bcl-2. Another study demonstrated that EVs loaded with miRNAs via electroporation significantly reduced the expression of MHC-I, MHC-II, and CD86 on dendritic cells, thereby inhibiting their maturation and promoting an immunosuppressive state [124].

In the context of TBI, astrocyte-derived exosomes enriched with miR-873a-5p (an miRNA upregulated in the brain post-TBI) have shown therapeutic potential by reprogramming microglia toward an anti-inflammatory M2 phenotype, thereby reducing neuroinflammation [125]. Furthermore, Kodali et al. reported that intranasal delivery of human mesenchymal stem cell-derived EVs (hMSC-EVs) 90 min post-TBI enhanced hippocampal neurogenesis and synaptic preservation in a dose-dependent manner, leading to sustained improvements in cognition and behavior [126]. Zhang et al. extended these findings by showing that bone marrow-derived MSC-EVs inhibited NF-κB p65 nuclear translocation, thereby suppressing neurotoxic A1 astrocyte activation, attenuating neuroinflammation, and promoting neuronal regeneration in SCI models. With their small size, ability to cross the blood–brain barrier, broad tissue distribution, and compatibility with multiple administration routes (e.g., intravenous, intranasal), EVs hold great promise as both biomarkers and vehicles for targeted drug delivery. Advances in nanotechnology further strengthen their translational potential in neurotrauma therapy [127].

Collectively, microbiota, probiotic, and stem cell-derived EVs may act synergistically, offering a versatile class of modulators and therapeutic agents with the potential to attenuate neuroinflammation, promote neuronal repair, and enhance functional recovery following neurotrauma.

The therapeutic potential of MEVs and PEVs depends on both their natural cargo and advances in engineering and delivery techniques. They can be enriched with endogenous molecules or loaded with exogenous agents and administered via intranasal, intravenous, or oral routes, making them versatile vectors for drug delivery and modulation of post-TBI neurological disorders. However, clinical translation remains challenging, including the complexity, cost, and need for standardized isolation and purification to ensure efficacy while minimizing adverse immune effects.

## 6. MEVs as Potential Therapeutic Agents and Precision Medicine Tools in TBI

### 6.1. Limitations of the Current Diagnostic Tools for TBI

Computed tomography (CT) and magnetic resonance imaging (MRI) are standard imaging modalities for assessing acute moderate to severe TBI, particularly in patients with a Glasgow Coma Scale (GCS) score below 13. However, both techniques often fail to detect subtle neuropathological changes associated with mild TBI (mTBI). Although MRI is more sensitive than CT for identifying diffuse axonal injury (DAI), it may appear unremarkable in early or mild cases. While these modalities offer improved sensitivity, they also present distinct challenges: Diffusion tensor imaging (DTI) can be confounded by transient alterations such as gliosis. Magnetic resonance spectroscopy (MRS) lacks standardized clinical protocols. Functional MRI (fMRI) results are often inconsistent due to inter-individual variability in neural activation patterns. ^18^F-fluorodeoxyglucose PET (^18^F-FDG PET) shows non-specific metabolic changes that are susceptible to modulation by neuroplasticity and vascular dynamics [127]. No current imaging technique provides sufficient sensitivity or specificity for independent diagnosis or prognosis in mild or chronic TBI, highlighting the need for novel biomarkers and artificial intelligence-integrated imaging to improve assessment of injury progression and treatment response [127]. In this context, EVs have gained attention as promising diagnostic and prognostic tools.

### 6.2. Extracellular Vesicles (EVs) as Complementary Biomarkers and Therapeutic Agents in TBI

EVs, including exosomes and microvesicles, have emerged as promising, minimally invasive tools for the diagnosis, monitoring, and potential treatment of TBI. Originating from various brain cell types, EVs encapsulate bioactive molecular cargo, including proteins, lipids, and RNAs. Of particular interest are microRNAs (miRNAs) like miR-124-3p and miR-155, which regulate neuroinflammatory responses. While miR-124-3p supports neuroprotection and microglial deactivation, miR-155 is linked to inflammation.

EVs possess several advantages as diagnostic tools: they can cross the BBB, remain stable in peripheral circulation, and retain molecular signatures reflective of their cellular origin. Temporal shifts in EV miRNA content allow for dynamic monitoring of TBI progression, enhancing their utility in both acute and chronic phases of injury [128].

Additionally, EVs serve as carriers of pathognomonic proteins, including tau, amyloid-β, and UCHL1, facilitating the detection of TBI severity and chronic traumatic encephalopathy (CTE) risk. The integration of multi-omics profiling combined with machine learning algorithms has further enabled biological subtyping, prognosis estimation, and personalized diagnostics based on EV signatures.

Beyond their diagnostic value, cell-derived EVs, especially those from mesenchymal stem cells (MSCs), neurons, astrocytes, and microglia, are being actively investigated for their therapeutic use. These vesicles deliver neuroprotective miRNAs such as miR-5121, miR-216a-5p, and the miR-17-92 cluster, supporting neurogenesis, synaptic repair, and anti-apoptotic pathways.

Engineered EVs carrying therapeutic molecules (e.g., Bcl-2, catalase, or brain-derived neurotrophic factor) have shown efficacy in reducing oxidative stress and inflammation in preclinical TBI models. The role of MEVs in TBI is still emerging; however, the success of host-derived EV therapies provides a strong rationale and mechanistic parallel for investigating MEVs. Collectively, these findings highlight the important role of MEVs as promising tools for precision medicine in TBI management [129,130].

Recent research has also paid attention to MEVs, which may exert parallel influences on brain function through gut–brain signaling pathways. These vesicles represent an emerging frontier in understanding systemic influences on brain injury and recovery.

### 6.3. Microbiota-Derived Extracellular Vesicles in Gut–Brain Signaling and Diagnosis

**Figure 1:**
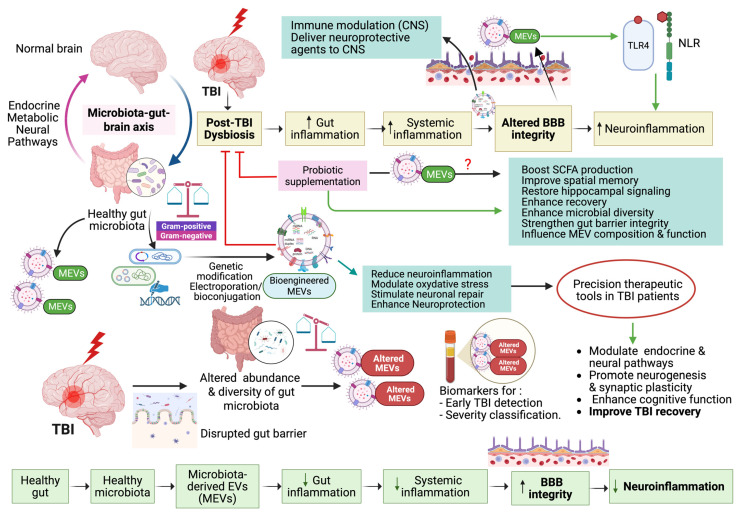
The potential role of microbial extracellular vesicles (MEVs) in the bidirectional communication via the gut microbiota–brain axis (GMBA) in TBI. Created in BioRender. Benameur, T. (2025) https://BioRender.com/7ls0h4q.

MEVs, secreted by both G+ and G- bacteria, are emerging as key mediators of microbiota–host communication. Their molecular stability and systemic accessibility position them as promising future diagnostic tools. These vesicles carry diverse bioactive molecules, including proteins, lipids, nucleic acids, and metabolites, and are capable of crossing the intestinal barrier and entering systemic circulation, and even reaching the brain via the systemic circulation or vagus nerve. Their lipid bilayer protects the MEV contents from enzymatic degradation, enhancing stability and their diagnostic reliability. Recent studies have shown that MEVs exhibit species-specific effects: *Akkermansia muciniphila* MEVs stimulate serotonin release, while *Lactobacillus plantarum* MEVs produce antidepressant-like effects in murine models. Similarly, *Bacteroides fragilis* releases MEVs that carry neurotransmitters like GABA and histamine, reinforcing their role in modulating neural signaling. Importantly, given that MEV cargo varies depending on microbial species and host context, they offer precision medicine potential by enabling personalized diagnostics or therapeutic strategies based on host–microbiota profiles [50,99]. Additionally, Banerjee et al. emphasize the broader implications of gut microbiota and their metabolites in regulating neurodevelopment and contributing to neurodegenerative disease processes, further emphasizing the relevance of microbial products in CNS health and disease mechanisms [131]. As illustrated below in Figure 1, MEVs play a vital role in mediating bidirectional communication through the gut microbiota–brain axis (GMBA) after TBI. MEVs can cross biological barriers, modulate neuroinflammation, and influence brain–gut signaling pathways. They also serve as potential accurate biomarkers for early TBI detection and severity classification. They can also enhance recovery by regulating immune responses and restoring gut–brain homeostasis.

### 6.4. Microbiota-Derived Extracellular Vesicles as Neurotherapeutic Agents: Mechanisms and Translational Potential

Emerging evidence highlights the neuroprotective potential of MEVs in a range of neurological disorders, including ischemic stroke, Alzheimer’s disease, Parkinson’s disease, and depression. Probiotic-derived MEVs, in particular, have been shown to reduce neuroinflammation, regulate oxidative stress, and modulate neurotransmitter signaling pathways. To enhance the specificity and therapeutic efficacy of these vesicles, MEVs can be bioengineered using two strategies: (1) genetic modification of the parent bacterial strain via tools such as CRISPR/Cas9 or plasmid vectors and (2) post-isolation methods, including electroporation or bioconjugation. These bioengineered vesicles offer a flexible and safe system for delivering neuroprotective agents directly to the CNS, without the need for administering live bacterial cells. As a result, MEVs show strong potential as non-cell-based, CNS-targeted delivery systems supporting their use in precision therapeutics [103]. In addition to their direct neuroprotective functions, MEVs are implicated in the broader context of microbiota–gut–brain axis (MGBA) interactions. Sun et al. have reported that brain injury is associated with chronic alterations in gut microbiota composition, which may exacerbate neurological dysfunction. This bidirectional interaction suggests a potential role for gut microbiota-derived extracellular vesicles (GMEVs) in mediating brain injury outcomes, particularly in conditions such as ischemic stroke and TBI. While direct experimental evidence for MEV-based interventions in TBI remains limited, their established roles in neuroinflammation, oxidative stress modulation, and supporting neural repair across CNS disorders make them strong candidates due to their therapeutic potential [104].

Their stability, bioactivity, and ability to mediate gut–brain signaling warrant further exploration as novel interventions for brain injury. Furthermore, adjunctive microbiota-modulating strategies such as probiotic supplementation and intermittent fasting (IF) may indirectly influence MEV-related pathways. For instance, Amaral et al. demonstrated that administration of *Lactobacillus helveticus* and *Bifidobacterium longum* in a murine model of TBI reversed injury-induced dysbiosis, improved spatial memory, and partially restored hippocampal signaling, supporting recovery along the gut–brain–liver axis. Although MEVs were not directly assessed in that study, such interventions may enhance microbial diversity, boost short-chain fatty acid (SCFA) production, and strengthen gut barrier integrity factors that influence MEV composition and function [132].

Acting through the microbiota–gut–brain axis (MGBA), these microbiota-targeted therapies have been shown to modulate immune, endocrine, and neural pathways, promoting neurogenesis, synaptic plasticity, and cognitive function, while concurrently reducing oxidative stress and inflammation [133]. Together, these findings underscore the importance of MEVs and microbiota-modulating strategies as innovative avenues for CNS repair and highlight their translational relevance for brain injury and related disorders.

### 6.5. Role of Artificial Intelligence and Multi-Omics Integration in TBI Decoding and Neurotrauma Precision

TBI is not a single disease, but a heterogeneous spectrum of conditions resulting from various forms of damage or trauma to the brain. This heterogeneity presents a significant challenge for diagnosis, prognosis, and therapeutic development. Recent advances in omics technologies, such as genomics, proteomics, transcriptomics, and metabolomics, have considerably enhanced our understanding of the molecular complexity underlying TBI.

These approaches capture dynamic biological responses over time, from acute metabolic changes to longer-term gene expression alterations [134,135]. When combined with neuroimaging and clinical data, machine learning facilitates the identification of molecular subtypes and predictive biomarkers, offering a system-level perspective of TBI pathophysiology. For example, metabolomics reveals disruptions in lipid metabolism and oxidative stress, while proteomic and transcriptomic data highlight CNS-specific markers and regulatory miRNAs linked to inflammation and degeneration [133]. Despite existing challenges such as inter-individual variability, sample heterogeneity, and the requirement for large well-characterized cohorts, AI-driven multi-omics approaches are increasingly being recognized for their potential to improve diagnostic precision and individualized risk stratification in TBI [134,135].

**Figure 2:**
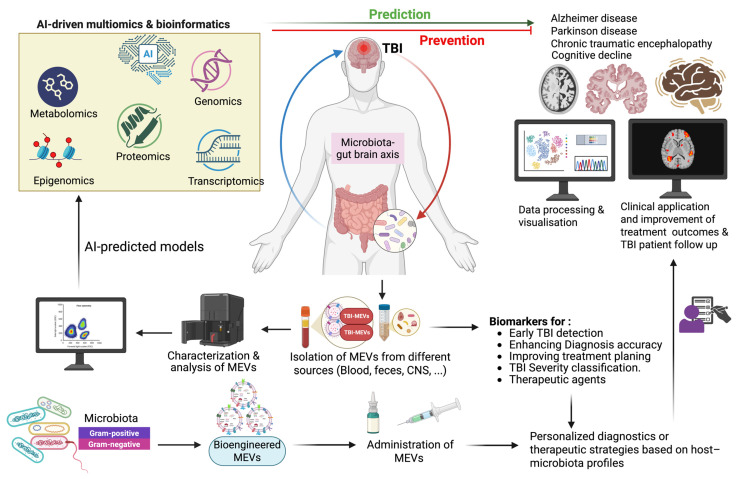
Microbiota-derived extracellular vesicles as precision biomarkers and neurotherapeutic agents in TBI. Targeting TBI with bioengineered MEVs to reach personalized diagnostic and enhance therapeutic strategies based on host-microbiome profiles. Multi-omics, encompassing metabolomics, proteomics, transcriptomics, epigenomics, and genomics, is a layered system of tools to understand TBI and its links to neurodegenerative diseases. Created in BioRender. Benameur, T. (2025) https://BioRender.com/b5vhjkf.

Figure 2 illustrates how AI-driven multi-omics predict, prevent, and respond across the post-injury timeline. It emphasizes their capacity to reveal critical biochemical changes and mechanistic links between TBI and long-term neurodegenerative outcomes, including Alzheimer’s disease, Parkinson’s disease, and chronic traumatic encephalopathy, and their associated cognitive impairment [135]. It also highlights the personalized diagnostic and therapeutic potential of bioengineered MEVs based on the host profile. Such an approach adds significant value not only to the diagnostic accuracy but also to the precision medicine in TBI patients by enhancing diagnostic accuracy and optimizing treatment outcomes.

Looking forward, an important frontier involves incorporating MEVs into these integrative frameworks. MEVs are emerging as systemic mediators of gut–brain communication and may influence neuroinflammatory and neurodegenerative cascades following TBI. Leveraging machine learning to analyze MEV profiles within multi-omics datasets could significantly enhance our ability to decode gut–EV–brain interactions and refine predictive models of TBI outcomes.

## 7. Conclusions and Future Directions

TBI remains a major public health problem due to its global prevalence, complex pathophysiology, and the lack of efficient therapeutic strategies. Despite the advanced understanding of the mechanisms underlying TBI, current clinical approaches remain insufficient in addressing its long-term cognitive, emotional, and neurological consequences. The heterogeneity in patient response and disease trajectory further complicates treatment, underscoring the urgent need for reliable biomarkers and targeted therapeutic interventions. Given the central role of the gut–brain axis in modulating neuroinflammation and systemic immune responses following brain injury, MEVs have emerged as promising tools with multifaceted roles in diagnosis, prognosis, and therapy. MEVs, secreted by both G+ and G- bacteria, are stable, systemically accessible, and able to cross the intestinal barrier to reach the CNS. Moreover, MEVs can also cross the BBB and transport microbial signals, including proteins, lipids, and RNAs, that influence neuronal survival, synaptic plasticity, and inflammatory responses.

MEVs have demonstrated the potential to serve as sensitive biomarkers for early TBI detection and severity classification. They also hold therapeutic promise through their capacity to modulate neuroinflammation and promote neural repair. Their use could pave the way for personalized interventions that account for an individual’s microbiome profile, systemic immune status, and injury characteristics.

Looking to the future, the integration of multi-omics technologies such as transcriptomics, proteomics, and metabolomics combined with artificial intelligence (AI) and machine learning algorithms is expected to unlock the full diagnostic and therapeutic potential of MEVs. These integrative platforms can identify complex molecular patterns, classify biological subtypes of TBI, and predict outcomes with greater precision. Moreover, AI-driven approaches will also aid in the real-time analysis of MEV-derived signatures, allowing for rapid, scalable, and clinically meaningful decision-making. This holds promise not only for deepening our mechanistic understanding of TBI but also for translating that knowledge into personalized and timely interventions.

To maximize the therapeutic and diagnostic potential of microbial extracellular vesicles (MEVs), we propose that future research should prioritize the following areas:

To maximize the diagnostic and therapeutic potential of MEVs, we propose that future research should prioritize the following areas:

- Standardizing protocols for MEV isolation, characterization, and quantification;
- Establishing large-scale longitudinal studies to validate MEV-based biomarkers across TBI subtypes;
- Exploring MEV engineering for targeted drug delivery and modulation of gut–brain axis signaling;
- Integrating MEV data into AI-powered clinical decision support systems to enhance diagnostic accuracy and treatment planning;
- Investigating the interaction between MEVs and other systemic factors (e.g., immune responses, metabolic changes) to develop comprehensive, systems-level models of TBI.

In conclusion, MEVs represent a transformative tool at the intersection of microbiology, neuroscience, and precision medicine. Their integration into clinical practice, supported by AI and multi-omics platforms, could provide an accurate method for the diagnosis, monitoring, and treatment of TBI, moving us closer to individualized and effective patient care.

## Figures and Tables

**Table 1:** Effects of MEVs on the pathogenesis of certain neurodegenerative disorders.

Neurological Disorders	Origin of MEVs	Effects	Reference
Alzheimer Disease	Commensal gut microbiota	Increase microglial activation Increase Aβ plaque accumulation	[105]
	*Helicobacter* *pylori*	Exacerbation of amyloid-β pathology and cognitive decline Accelerate the development of AD through OMV and C3-C3aR signaling	[106]
	*Porphyromonas gingivalis*	Gingipains, LPS induce inflammation Memory impairment Increase the expression of proinflammatory factors Reducing the expression of BDNF receptor, claudin-5, and N-methyl-D-aspartate	[107]
		Decreases the tight junction-related gene expression ZO-1, occludin, claudin-5, and occludin protein expression in the hippocampus Activates both astrocytes and microglia and increases IL-1β, tau phosphorylation at Thr231 site, and inflammasome-related protein NLRP3 expression Causes memory dysfunction, neuroinflammation	[108]
		Gingipains present in the region around the cerebral ventricles, choroid plexus, and ventricular ependymal cells in mice increased the gene expression of proinflammatory cytokines in HMC3 cells in a gingipain-dependent manner.	[109]
	*Lactobacillus johnsonii*	Increase in Clostridium genus bacteria in the small intestine	[110]
Parkinson Disease	*Escherichia coli*	Enhanced DAPK1 expression to enhance Ser129-phosphorylated α-syn	[116]

## Data Availability

Data sharing is not applicable to this article.
